# Effectiveness of the physical activity promotion programme on the quality of life and the cardiopulmonary function for inactive people: Randomized controlled trial

**DOI:** 10.1186/1471-2458-13-127

**Published:** 2013-02-12

**Authors:** Rocío Martín-Valero, Antonio Ignacio Cuesta-Vargas, María Teresa Labajos-Manzanares

**Affiliations:** 1 Department of Physiotherapy, Faculty of Health Sciences, University of Málaga, Av/ Martiricos s/n, Málaga 29009, Spain; 2 School of Clinical Science at Queensland University of Technology, Level 6, O Block, D Wing, Kelvin Grove, Brisbane, Australia

**Keywords:** Promotion physical activity, Cardiopulmonary, Quality of life

## Abstract

**Background:**

The purpose is to assess cardiopulmonary function outcomes and quality of life values in inactive people that participated in the Physical Activity Promotion Programme (PAPP) against the control group that did not perform this program.

**Method:**

A total of 100 subjects of both genders participated in the randomized controlled trial with systematic random sampling; all were aged 55 and older, from Torremolinos, Spain. Participants either received (n = 50) the PAPP for 60 minutes, twice a week during three months or (n = 50) they received health education. The effectiveness of the intervention was measured by general state of health the Short Form 12 health survey questionnaire, and the quality of life was determined with the EuroQoL-5D questionnaire. Cardiopulmonary function was measured with a spirometry and a walking test according to the Bruce protocol.

**Results:**

This pilot study had a significant impact on the quality of life (p = 0.05) in men, which increased. However, the quality of life in women did not improve. The average changes in the lung and cardiovascular function was not significant between groups.

**Conclusions:**

Changes in the quality of life measured with EQ-5D in the group of men who carried out the PAPP were statistically significant when comparing between groups. However changes in cardiopulmonary function were not as relevant when comparing between groups. There was a significant effect within each group in the pulmonary outcomes of values in men, within the experimental group.

**Trial registration:**

Developed by the University of Málaga. ClinicalTrials.gov ID: NCT01172483.

## Background

Prospective observational studies have suggested that inactive people have more death risk due to no specific cause and from specific diseases (e.g., cardiovascular disease, diabetes, obesity and others) associated with physical inactivity [1]. There is evidence that regular physical activity contributes to the primary and secondary prevention of several chronic diseases and is associated with a reduced risk of premature death [2].

Health-related quality of life is an important measure of the effect of an intervention program on cardiovascular disease [3]. There are studies in inactive people which have observed a decrease in lung function [4]. Forced expiratory volume in one second (FEV_1_) was used in the cohort study of Buffalo as a predictor of survival and as a tool to assess the overall health of the population [5].

Epidemiological studies have showed that physical activity has a protective effect towards cardiovascular disease [6]. The physical activity helps to regulate the blood pressure [7]. There are previous studies about the physician’s role in promoting physical activity [8]. However, it is necessary to clarify the power of promoting physical activity for inactive people in the Primary Healthcare Centers.

The aim of the current study was to evaluate the effectiveness of the Physical Activity Promotion Programme (PAPP) on the quality of life and cardiopulmonary outcomes for the inactive subjects compared with people receiving education and advice in Primary Healthcare Centers. Therefore this study compares the observed changes in both the quality of life and cardiopulmonary outcomes for people who participated in the PAPP and those that did not carry out it.

## Methods

### Study design and participants

The design was a randomized controlled pilot clinical trial.

One hundred people from Primary Healthcare Centers in Málaga begun this research and seventy-five subjects completed the study. The age of the participants of both genders ranged between 57 and 69.

### Inclusion and exclusion criteria

The General Practitioner selected the participants who co-operated with the study. The criteria to be included in the study were inactive people, not engaged, with moderate physical activity for at least thirty minutes, five times a week [9]. Participants also had to have three or more of the following cardiovascular risk factors: increased blood pressure 140/90, smoking, cholesterol above 230 mg / dl, a family member that had suffered a heart attack before the age of 55 if male or before the age of 65 if female and an obese or overweight (more than 8 kg) insulin-dependent diabetic [10].

The criteria to be excluded from participation in the study were the following: infectious processes, malignancy, metastasis, osteoporosis, inflammatory arthritis or fractures, cognitive impairment due to any cause [11].

### Randomization

The people who would participate in this study were randomized systematic procedures during the recruitment period. Random sequence generation was performed; people had to take a closed envelope from a box in order to form part of the intervention group or the control group. All the subjects who met the inclusion criteria had to carry out an initial evaluation before starting the procedure and at the end of twelve weeks the same procedure was carried out. The researcher, who did the evaluation, did not know what subjects would be assigned to each group.

### Procedure

#### Physical activity promotion programme (PAPP)

The intervention group (IG) performed a PAPP twice a week for twelve weeks, following the criteria of the American College of Sports Medicine [9] in the Sports and Physical Medical Center in Torremolinos. An assigned professional health specialist carried out the procedure between October and March of 2010 and 2011. Each session lasted 60 minutes, and all protocols were developed for progressive intensity, depending on each person. The sessions were organized at the early stage of heating, followed by the aerobic phase and the cooling-stretch or final phase.

### Control group (CG)

The control group received an educational health leaflet containing advice from Primary Healthcare Centers in Málaga, and continued with their daily routine activities. Subjects were assessed on the outcome measures at baseline, at twelve weeks follow-up by an independent blind assessor, at the Sports and Physical Medical Center in Torremolinos, Spain.

### Outcome measures

The primary outcomes were the following: General Health Questionnaire determined with the Short Form-12 Health Survey (SF-12) and Health-related Quality of Life (HRQOL), determined with the EuroQoL-5D (EQ-5D).

The SF-12 questionnaire is a shortened version of the SF-36, and has a reliability coefficient of 0.97 [12]. We report the results of eight general health dimensions: physical functioning, physical role, body pain, general health, vitality, social functioning, emotional role and mental health [13]. These eight dimensions can also be used to generate both physical and mental health summary scores [12]. The SF-12 questionnaire will prove to be a practical alternative to the SF-36, in order to measure the overall health of the population, because of the high degree of correspondence between estimated physical and mental health measures, using the SF-12 and SF-36 questionnaires [14].

The EQ-5D has five domains: mobility, self-care, usual activities, pain, and anxiety/depression [15]. Each domain has three possible levels indicating; no problems, moderate problems or severe problems [16]. The EQ-5D valuation questionnaire comprises a visual analogue scale which was not included in this research. It has shown to be a acceptable and valid tool, with an average estimation of 0.87 [17].

The secondary outcomes were cardiopulmonary. The cardiac outcomes were at resting heart rate, and achieved at the end of the test. Subjects were questionnaire about the rate perceived effort (RPE) several times during the exercise test [18]. The pulmonary outcomes were the Tiffenau index, which is the ratio between forced expiratory volume in one second and forced vital capacity (FEV_1_/FVC), forced vital capacity (FVC) and forced expiratory volume in one second (FEV_1_). The values were expressed in absolute terms in milliliters and as percentage of theoretical value for individuals of the same age, weight and height in the reference population.

### The exercise test

Subjects performed an exercise test on a treadmill according to the Bruce protocol [19]. The sub maximal protocol was used for the exercise test in which the subject would be over 85% of the maximum frequency. The test was brought to an end when the subject reached the maximum achievable intensity, taking into account the following criteria: the theoretical maximum heart rate according to age, usually calculated as two hundred twenty minus age in years.

Participants were asked to identify their perception of perceived exertion every thirty seconds and end the test with the scale of effort adapted by Borg (RPE) from zero to ten points [18]. The scale of the effort perception was defined as the subjective intensity of effort, stress, discomfort and / or fatigue you had felt during exercise [18].

The heart rate was measured at the beginning and at the end of the exercise test, which was called “HRf”, and was obtained at the end of the running test on the treadmill.

### Spirometry

The simple spirometry was used to measure lung outcomes with pneumotachograph Fleisch DATOSPIR 120 according to the SEPAR’s criteria [20]. Three maneuvers were performed in order to get only the best values for the analysis. The values are determined with forced vital capacity (FVC), forced expiratory volume in one second (FEV_1_), Tiffenau index which is the ratio between forced expiratory volume in one second and forced vital capacity (FEV_1_/FVC). The values are expressed in absolute terms in milliliters and as percentage of the theoretical value for individuals of the same age, weight and height in the reference population.

### Data collection

All participants received information about the research and gave their written consent before participation. We performed a general clinical interview which included an exercise test and a spirometry.

### Data management and analysis

All analyses were conducted using SPSS version 17.0. Kolmogorov-Smirnov tests were used to analyze the normality of the data distribution. Student’s T tests for relational samples were performed to get the differences within each group after the intervention. We also used Student’s *T* test for independent samples to get the inter-group effect of this intervention.

### Sample size

The sample size was calculated with an alpha error of 0.05, a power of 0.80 and a beta risk of 0.20 according to the effect size of EQ-5D that it was made according to Cohen’s criteria [21]. One hundred subjects took part in this research against 60 individuals are needed in a priori estimation: 30 individuals in the IG and 30 in the CG.

### Evaluation of clinical relevance

Cohen’s criteria were taken into account in the analysis of the effect size values, his criteria determine that values below 0.2 are considered to have no effect, those between 0.2 and 0.5 a small effect, between 0.5 and 0.8 a medium effect, and those above 0.8 are considered to have a huge effect [21].

### Ethics

This study was authorized by the Ethics and Research Committee of the “Costa del Sol” Health District. All participants gave written informed consent, confidentiality and anonymity were also preserved at all times and the principles of the Declaration of Helsinki were respected.

## Results

The sample consisted of thirty-one men and forty-four women. The subjects mean aged was 62.28 ± 6.9 years**.** Gender difference is reflected in all data. The initial characteristic of the participants are shown in Table 1. Regarding the systolic and diastolic blood pressure at rest, the initial values of systolic blood pressure were greater than 140 mm Hg (Table 1). The descriptive values of the changes within each group**,** obtained in the self-reported questionnaires and the cardiopulmonary outcomes by gender difference (Table 2).

**Table 1:** Initial characteristic according the gender difference

	**Control group**		**Intervention group**	
	**Men mean (CI)**	**Women mean (CI)**	**Men mean (CI)**	**Women mean (CI)**
age (years)	64.25 (59–69)	62.82(60–65)	60.50 (57–63)	63.26(60–66)
Weight (Kg)	93.29(79–106)	78.50(71–85)	87.44 (77–97)	78.21(71–85)
Height (metre)	1.68(1.64–1.72)	1.58(1.5–1.60)	1.67 (1.62–1.74)	1.56(1.54–1.59)
SPB (mm Hg)	156(143–168)	133(127–140)	144(134–154)	131(125–137)
DPB (mm Hg)	83(76–91)	78.91(75–82)	79(69–89)	80.21(76–85)
HRr (B/min)	73(66–80)	76.18(72–80)	75.17(68–82)	80.04(74–86)
HRmax (B/min)	132(127–136)	133.43(131–135)	134(130–138)	132.57(129–136)
HRf (B/min)	122(114–130)	125(118–132)	124(115–132)	123.21(115–131)
RPE	5.75(4.7–6.8)	5.27(4.50–6.04)	5.28(4.44–6.11)	5(4.42–5.58)
FVC (L)	3.78(2.9–4.6)	2.76(2.21–3.3)	3.88(3.14–4.62)	2.54(2.22–2.86)
FEV_1_ (L)	2.55(1.94–3.16)	1.68(1.32–2.03)	2.44(1.99–2.9)	1.77(1.51–2.02)
FEV_1_/FVC (%)	66.59(57–76)	68(59–78)	65.61(57–74)	71(63–79)
EQ-5D (0–1)	0.57(0.38–0.75)	0.59(0.48–0.72)	0.58(0.36–0.76)	0.53(0.40–0.67)
Physical role (0–100)	46(40–51)	44.32(40–49)	48(43–53)	42.5(37–48)
Social (0–100)	26.27(26.3–26.3)	31.71(23–40)	31.3(20.22–42.4)	25.15(18–32)
Vitality (0–100)	52.8(46–60)	49(43–55)	46.1(40–52)	51(44–57)
Mental (0–100)	34.06(26–42)	36.43(30–42)	37.5(30–44)	39(33–45)

CI: confidence interval.

SPB: systolic blood pressure.

%: percentage.

L: liter.

B/min: beats per minute.

HRr: heart rate on resting.

DPB: diastolic blood pressure.

HRf: heart rate at the end.

HRmax: maximum heart rate.

RPE: rate perceived effort.

**Table 2:** Changes within each group obtained in the cardiopulmonary outcomes and the questionnaires according gender difference

	**Control group**		**Intervention group**	
	**Men mean (CI)**	**Women mean (CI)**	**Men mean (CI)**	**Women mean (CI)**
Weight (Kg)	1.95(0.47-3.44)**	−0.63(−2.19-0.92)	−0.05 (−3.13-3.02)	−0.50(−1.82-0.82)
SPB (mm Hg)	13.08(0.53-25.63)*	−3.13(−8.73-2.46)	−4.33(−20-11.44)	−4.65(−10.27-1.41)
DPB (mm Hg)	0.83(−4.83-6.49)	−0.04(2.40-2.31)	−4.50(−16-7.08)	1.43(−2.93-5.80)
HRr (B/min)	−3.75(−9.86-2.36)	−4.5(−8.45-(−0.54)*	−2.16(−5.80-1.47)	1.04(−2.64-4.72)
HRmax (B/min)	−0.58(−1.86-0.70)	−0.09(−0.32-0.142)	−0.42(−1.23-0.40)	−1.78(−4.62-1.05)
HRfinal (B/min)	4(−1.92-9.92)	1.72(−4.75-8.21)	1(−6.01-8.01)	3.82(−4.09-11.74)
RPE	0.83(−0.13-1.80)	0.91(0.22-1.59)**	0.22(−0.60-1.05)	0.39(−0.29-1.07)
FVC (L)	0.82(0.33-1.31)	0.38(−0.09-0.85)	0.73(0.18-1.27)**	0.10(−0.06-0.27)
FEV1 (L)	0.06(−0.38-0.51)	−0.02(−0.24-0.19)	0.11(−0.03-0.26)	−0.07(−0.23-0.08)
FEV1/FVC (%)	17.47(−28.01-(−6.95)	−1.22(−11.30-8.85)	−10.45(−18.6-(−2.23)**	5.17(−11.45-1.11)
EQ-5D (0–1)	−0.24(−0.43-0.043)*	0.065(−0.166-0.03)	0.02(−0.18-0.23)	0.14(−0.30-0.01)
Physical role (0–100)	0(−7.27-7.27)	3.43(−1.14-8.01)	1.16(−5.12-8.34)	4.29(−0.619-9.21)
Social (0–100)	10.09(−8.45-28.65)	1.12(−12.58-14.81)	10.09(−8.45-28.65)	8.07(−21.81-5.65)
Body Pain (0–100)	7.64(−0.46-15.75)*	8.33(0.95-15.72)*	5.09(−4.26-14.45)	5.09(−4.26-14.45)
General Health (0–100)	1.23(−2.69-5.16)	0.43(−3.18-2.32)	3.50(−7.58-0.58)	0.95(−3.29-1.37)
Vitality(0–100)	8.51(−1.67-18.70)	0(−5.11-5.11)	2.87(−5.15-10.90)	2.68(−5.32-10.69)
Mental(0–100)	2.03(−9.17-13.23)	3.04(−15.42-9.32)	1.74(−10.31-13.79)	6.70(−3.03-16.44)

**p = 0.01.

CI: confidence interval.

SPB: systolic blood pressure.

RPE: rate perceived effort.

*p = 0.05.

HRr: heart rate on resting.

HRf: heart rate at the end.

HRmax: maximum heart rate.

B/min: beats per minute.

%: percentage.

FEV_1_: forced expiratory volume.

FEV_1_/FVC: Tiffenau index.

FVC: forced vital capacity.

L: liter.

In our study, the changes between both the experimental and the control group in the self-reported questionnaires found the range of EQ-5D of the male population with a difference between groups in the effect size value of 0.05 (p = 0.05), whereas women did not improve their quality of life. The changes between both the experimental and control group in the cardiopulmonary function outcome for the FEV_1_/FVC value did not show statistically significant changes in the experimental group, which the mean FEV_1_/FVC value for both men and women was (−10.45 vs. 5.17%) respectively (Table 2). The men of the control group showed a greater negative effect (−17.48 vs. -1.23%), compared to women with a smaller negative effect (Table 2).

## Discussion

Quality of life improved significantly for inactive subjects who carried out the PAPP (p = 0.05). Besides, pulmonary function outcomes FVC and FEV_1_/FVC values found statistically significant differences within experimental group. However, these differences were not found within the control group. Other clinical trials with community intervention programs have increased the level of physical activity, without improving the level of the quality of life [22]. In contrast, others have improved the quality of life without changing the level of physical activity [23]. Statistically differences were observed for people with depression after the DeLLITE program of physical activity in the quality of mental health, measured with the SF-36 questionnaire [24].

In previous research, the criteria established for the minimum important differences of the ranged of EQ-5D from 0.033 [25] to 0,074 [26]. In the current study, the male population was within this range of EQ-5D with a difference between groups in the effect size value of 0.05 (p = 0.05), whereas women did not improve their quality of life. In cardiac patients an effect size of 0.31, (p = 0.001) was observed in the quality of life with a positive statistical significance [27]. However, a cross-sectional study showed a decreased quality of life value with respect to the EQ-5D questionnaire, in the people aged between 66–79 years with sedentary lifestyles [28]. Furthermore, it is necessary to reach the consensus about the clinically important difference for EQ-5D.

According to the general health status which was determined with the SF-12 questionnaire there were no statistically significant changes between groups, after the intervention of the PAPP, as the current study has shown. However, other studies showed improved cognitive function (effect size 1.17) in people who performed physical activity [29]. Like as in other studies, they found that active subjects had fewer psychological problems than inactive subjects [30]. Statistically also changes in the SF-36 questionnaire had been observed after performing a program of physical activity in water during two months. These improvements were obtained in all the fields of the quality of life, except in the emotional role and in general health [31].

Regarding the systolic and diastolic blood pressure, we did not observe statistically significant differences in the effect size on diastolic blood pressure after the procedure. In contrast, the effect size for cardiac subjects showed statistically better improvement (p = 0.01) [27]. In a recent meta-analysis, both resistance training programs and isometric hand grip could help decrease blood pressure [32]. Besides, resistance programs could decrease the risk of cardiovascular disease [33].

There were a slight decrease in systolic blood pressure (SDP) at rest concerning the same value in both men and women in the experimental group (−4.33 vs. -4.65 Mm Hg). However, men in the control group showed an increase systolic blood pressure (13.09 vs. -3.13 Mm Hg, p = 0.05) compared with women who showed a decrease in systolic blood pressure. In contrast, people with mild hypertension found a significant decrease in systolic blood pressure, as a result of performing a program of low intensity running [34].

Respect to diastolic blood pressure (DBP) not statistically significant changes were found within each group. There was a decrease in DBP in men in the experimental group (−4.5 vs. 1.43 mm Hg) versus a slight increase in DBP in women (Table 2). However, people with hypertension got statistically significant decrease after receiving a program of low intensity exercise [34]. Women with metabolic syndrome also got decrease both blood pressures when performed a program of low intensity [35]. Besides, a decrease in blood pressure has been observed after performed a program of moderate intensity resistance exercises [36].

According to the cardiac outcomes not statistically significant changes were found between both the experimental and control group. However, we found statistically significant changes in resting HR in the control group of women. The mean HR value was (−3.75 vs. -4.5, p = 0.05) in men and women respectively. On the other hand, statistically significant changes were not observed in the experimental group (Table 2). There are studies that found differences in the cardiac function depending on the amount of body fat of the subject [37]. The Fit & Firm program showed significant improvement in heart rate [38]. It could be possible that the cardiac function response depends on a mix of genetic factor and the intensity of training.

The pulmonary outcomes except FEV_1_ improved significantly (p < 0.01) in men of the experimental group. In a recent study, all pulmonary function parameters except FEV_1_/FVC improved significantly (p < 0.0001) in both yoga and swimming groups [39]. Besides, better pulmonary functions in subjects performing yoga as well as swimming are documented [4,40]. Statistically significant changes in pulmonary function of FEV_1_/FVC and FEV_1_ values in people who performed regularly physical activity compared with sedentary people were shown [4]. Apart from that, the participants performed a four-week program who found statistically significant changes in FEV_1_ and FEV_1_/FVC (3.96 vs. 0.96, p = 0.001), but there were no statistically significant changes in the FVC value (4.13, p = 0.43) [39]. This program included upper limb resistive exercises for thirty minutes, supplemented with ten minutes of breathing exercises [39]. Nevertheless, the program “Exercise on Prescription” not found effect on the pulmonary outcome in the physical inactive women in the Netherlands [41].

Type II error should take into account the outcomes which did not show effects due to lack of subjects. Future studies will be necessary to find the reason to explain the difference in FVC and FEV_1_/FVC outcomes between men and women [5].

## Conclusions

Changes statistically significant in the quality of life measured with EQ-5D in the group of men who carried out the PAPP when comparing between groups. However changes in cardiopulmonary function were not as relevant when comparing between groups. On the contrary, there was a significant effect within each group in the pulmonary outcomes of values in men, within the experimental group.

To ensure the quality of this randomized clinical trial the guide developed by the CONSORT statement (Consolidated Standards of Reporting Trials) [42] has been followed. ClinicalTrials.gov ID: NCT01172483.

## Competing interests

The authors declare that they have no competing interests.

## Authors’ contributions

All authors have made significant contributions to the article. AICV coordinated the project and contributed to the conception and design of this study. RMV and AICV were responsible of the acquisition, analysis and interpretation of data, both contributed to the screened articles and drafted the manuscript. AICV and MTL made substantial contributions to the critical revision of the paper. All authors read and approved the final manuscript.

## Pre-publication history

The pre-publication history for this paper can be accessed here:

http://www.biomedcentral.com/1471-2458/13/127/prepub

## References

[B1] Haskell WL Blair SN Hill JO Physical activity: health outcomes and importance for public health policy Prev Med 2009 49 4 280 282 10.1016/j.ypmed.2009.05.002 19463850

[B2] Warburton DE Nicol CW Bredin SS Health benefits of physical activity: the evidence CMAJ 2006 174 6 801 809 16534088 10.1503/cmaj.051351 PMC1402378

[B3] Lam CL Lauder IJ The impact of chronic diseases on the health-related quality of life (HRQOL) of Chinese patients in primary care Fam Pract 2000 17 2 159 166 10.1093/fampra/17.2.159 10758080

[B4] Prakash S Meshram S Ramtekkar U Athletes, yogis and individuals with sedentary lifestyles; do their lung functions differ? Indian J Physiol Pharmacol 2007 51 1 76 80 17877296

[B5] Schunemann HJ Dorn J Grant BJ Winkelstein W Jr Trevisan M Pulmonary function is a long-term predictor of mortality in the general population: 29-year follow-up of the Buffalo Health Study Chest 2000 118 3 656 664 10.1378/chest.118.3.656 10988186

[B6] Lee IM Physical activity and cardiac protection Curr Sports Med Rep 2010 9 4 214 219 20622539 10.1249/JSR.0b013e3181e7daf1

[B7] Scher LM Ferriolli E Moriguti JC Scher R Lima NK The effect of different volumes of acute resistance exercise on elderly individuals with treated hypertension J Strength Cond Res 2011 25 4 1016 1023 20657311 10.1519/JSC.0b013e3181c70b4f

[B8] Brown WJ Individual or population approaches to the promotion of physical activity…is that the question? J Sci Med Sport 2006 9 1–2 35 37 discussion 38–9 16546446 10.1016/j.jsams.2006.02.005

[B9] Haskell WL Lee IM Pate RR Powell KE Blair SN Franklin BA Physical Activity and Public Health: Updated Recommendation for Adults From the American College of Sports Medicine and the American Heart Association Circulation 2007 116 9 1081 1093 17671237 10.1161/CIRCULATIONAHA.107.185649

[B10] Weng C Tu SW Sim I Richesson R Formal representation of eligibility criteria: a literature review J Biomed Inform 2010 43 3 451 467 10.1016/j.jbi.2009.12.004 20034594 PMC2878905

[B11] Van Spall HG Toren A Kiss A Fowler RA Eligibility criteria of randomized controlled trials published in high-impact general medical journals: a systematic sampling review JAMA 2007 297 11 1233 1240 10.1001/jama.297.11.1233 17374817

[B12] Ware J Kosinski M Keller SD A 12-Item Short-Form Health Survey: construction of scales and preliminary tests of reliability and validity Med Care 1996 34 3 220 233 10.1097/00005650-199603000-00003 8628042

[B13] Jenkinson C Layte R Development and testing of the UK SF-12 (short form health survey) J Health Serv Res Policy 1997 2 1 14 18 10180648 10.1177/135581969700200105

[B14] Gandek B Ware JE Aaronson NK Apolone G Bjorner JB Brazier JE Cross-validation of item selection and scoring for the SF-12 Health Survey in nine countries: results from the IQOLA Project, International Quality of Life Assessment J Clin Epidemiol 1998 51 11 1171 1178 10.1016/S0895-4356(98)00109-7 9817135

[B15] Herdman M Badia X Berra S EuroQol-5D: a simple alternative for measuring health-related quality of life in primary care Aten Primaria 2001 28 6 425 430 11602124 10.1016/S0212-6567(01)70406-4 PMC7684037

[B16] Papaioannou D Brazier J Parry G How valid and responsive are generic health status measures, such as EQ-5D and SF-36, in schizophrenia? A systematic review Value Health 2011 14 6 907 920 10.1016/j.jval.2011.04.006 21914513 PMC3179985

[B17] Jia H Lubetkin EI Estimating EuroQol EQ-5D scores from Population Healthy Days data Med Decis Making 2008 28 4 491 499 10.1177/0272989X07312708 18556640

[B18] Borg GA Psychophysical bases of perceived exertion Med Sci Sports Exerc 1982 14 5 377 381 7154893

[B19] Mahler DA Froelicher VF Miller NH York TD ACSM’s Guidelines for Exercise Testing and Prescription 1995 5 Baltimore, Md: Williams & Wilkins

[B20] Miller MR Hankinson J Brusasco V Burgos F Casaburi R Coates A Crapo R Standardisation of spirometry Eur Respir J 2005 26 2 319 338 10.1183/09031936.05.00034805 16055882

[B21] Cohen J Statistical power analysis for the behavioral sciences 1988 Available from: [ http://www.questia.com/PM.qst?a=o&d=98533106]

[B22] Kolt GS Schofield GM Kerse N Garrett N Oliver M Effect of telephone counseling on physical activity for low-active older people in primary care: a randomized, controlled trial J Am Geriatr Soc 2007 55 7 986 992 10.1111/j.1532-5415.2007.01203.x 17608869

[B23] Kerse N Elley CR Robinson E Arroll B Is physical activity counseling effective for older people? A cluster randomized, controlled trial in primary care J Am Geriatr Soc 2005 53 11 1951 1956 10.1111/j.1532-5415.2005.00466.x 16274377

[B24] Kerse N Hayman KJ Moyes SA Peri K Robinson E Dowell A Home-based activity program for older people with depressive symptoms: DeLLITE–a randomized controlled trial Ann Fam Med 2010 8 3 214 223 10.1370/afm.1093 20458104 PMC2866718

[B25] Sullivan PW Lawrence WF Ghushchyan V A national catalog of preference-based scores for chronic conditions in the United States Med Care 2005 43 7 736 749 10.1097/01.mlr.0000172050.67085.4f 15970790

[B26] Walters SJ Brazier JE Comparison of the minimally important difference for two health state utility measures: EQ-5D and SF-36D Qual Life Res 2005 14 6 1523 1532 10.1007/s11136-004-7713-0 16110932

[B27] Conn VS Hafdahl AR Moore SM Nielsen PJ Brown LM Meta-analysis of interventions to increase physical activity among cardiac subjects Int J Cardiol 2009 133 3 307 320 10.1016/j.ijcard.2008.03.052 18582959 PMC2702092

[B28] Kostka T Bogus K Independent contribution of overweight/obesity and physical inactivity to lower health-related quality of life in community-dwelling older subjects Z Gerontol Geriatr 2007 40 1 43 51 10.1007/s00391-006-0374-6 17318731

[B29] Angevaren M Aufdemkampe G Verhaar HJ Aleman A Vanhees L Physical activity and enhanced fitness to improve cognitive function in older people without known cognitive impairment Cochrane Database Syst Rev 2008 3 3 CD005381 18646126 10.1002/14651858.CD005381.pub3

[B30] Akbartabartoori M Lean ME Hankey CR The associations between current recommendation for physical activity and cardiovascular risks associated with obesity Eur J Clin Nutr 2008 62 1 1 9 10.1038/sj.ejcn.1602693 17342166

[B31] Saavedra JM De La Cruz E Escalante Y Rodriguez FA Influence of a medium-impact aquaerobic program on health-related quality of life and fitness level in healthy adult females J Sports Med Phys Fitness 2007 47 4 468 474 18091689

[B32] Cornelissen VA Fagard RH Coeckelberghs E Vanhees L Impact of resistance training on blood pressure and other cardiovascular risk factors: a meta-analysis of randomized, controlled trials Hypertension 2011 58 5 950 958 10.1161/HYPERTENSIONAHA.111.177071 21896934

[B33] Kodama S Saito K Tanaka S Maki M Yachi Y Asumi M Cardiorespiratory fitness as a quantitative predictor of all-cause mortality and cardiovascular events in healthy men and women: a meta-analysis JAMA 2009 301 19 2024 2035 10.1001/jama.2009.681 19454641

[B34] Hua LP Brown CA Hains SJ Godwin M Parlow JL Effects of low-intensity exercise conditioning on blood pressure, heart rate, and autonomic modulation of heart rate in men and women with hypertension Biol Res Nurs 2009 11 2 129 143 10.1177/1099800408324853 19150992

[B35] Roussel M Garnier S Lemoine S Gaubert I Charbonnier L Auneau G Influence of a walking program on the metabolic risk profile of obese postmenopausal women Menopause 2009 16 3 566 575 10.1097/gme.0b013e31818d4137 19057414

[B36] Fagard RH Cornelissen VA Effect of exercise on blood pressure control in hypertensive patients Eur J Cardiovasc Prev Rehabil 2007 14 1 12 17 10.1097/HJR.0b013e3280128bbb 17301622

[B37] Chantler PD Clements RE Sharp L George KP Tan LB Goldspink DF The influence of body size on measurements of overall cardiac function Am J Physiol Heart Circ Physiol 2005 289 5 H2059 H2065 10.1152/ajpheart.00022.2005 15964929

[B38] King AC Pruitt LA Phillips W Oka R Rodenburg A Haskell WL Comparative effects of two physical activity programs on measured and perceived physical functioning and other health-related quality of life outcomes in older adults J Gerontol A Biol Sci Med Sci 2000 55 2 M74 M83 10737689 10.1093/gerona/55.2.m74

[B39] Singh VP Jani H John V Singh P Joseley T Effects of upper body resistance training on pulmonary functions in sedentary male smokers Lung India 2011 28 3 169 173 10.4103/0970-2113.83971 21886949 PMC3162752

[B40] Gupta SS Sawane MV A comparative study of the effects of yoga and swimming on pulmonary functions in sedentary subjects Int J Yoga 2012 5 2 128 133 10.4103/0973-6131.98232 22869997 PMC3410192

[B41] Gademan MG Deutekom M Hosper K Stronks K The effect of exercise on prescription on physical activity and wellbeing in a multi-ethnic female population: A controlled trial BMC Public Health 2012 12 1 758 10.1186/1471-2458-12-758 22963588 PMC3490781

[B42] Atman DG Schulz KF Moher D Egger M Davodpff F Elbourne D CONSORT GROUP (Consolidated Standards of Reporting Trials) The revised CONSORT statement for reporting randomized trials: explanation and elaboration Ann Int Med 2001 134 8 663 694 11304107 10.7326/0003-4819-134-8-200104170-00012

